# Associations between social support and physical activity in postpartum: a Norwegian multi-ethnic cohort study

**DOI:** 10.1186/s12889-023-15507-z

**Published:** 2023-04-17

**Authors:** Karin Elisabeth Bennetter, Kåre Rønn Richardsen, Nina Køpke Vøllestad, Anne Karen Jenum, Hilde Stendal Robinson, Ibrahimu Mdala, Christin Wiegels Waage

**Affiliations:** 1 Institute of Health and Society, Department of General Practice, Faculty of Medicine, University of Oslo, Box 1130, Blindern, Oslo, 0318 Norway; 2 Institute of Health and Society, Department of Interdisciplinary Health Sciences, University of Oslo, Oslo, Norway; 3 Department of Rehabilitation Science and Health Technology, Faculty of Health Science, Oslo Metropolitan University, Oslo, Norway; 4 General Practice Research Unit (AFE), Institute of Health and Society, Department of General Practice, University of Oslo, Oslo, Norway

**Keywords:** Physical activity, MVPA, Social support, Postpartum, Family, Friends, Ethnicity

## Abstract

**Background:**

Social support is associated with higher self-reported physical activity (PA) in postpartum women, but it is unknown if similar association occur when using objective PA data. The aim was to explore the associations between social support and objectively recorded moderate-to-vigorous physical activity (MVPA) postpartum, and if associations differed across ethnic groups.

**Methods:**

We used data from 636 women who participated in the STORK Groruddalen cohort study (2008–2010). MVPA minutes/day in bouts of ≥ 10 minutes was recorded by SenseWear Armband™ Pro^3^ (SWA) over 7 days, 14 weeks postpartum. Social support for PA from family or friends was measured by a modified 12-item version of the Social Support for Exercise Scale. We used single items, family support mean score (6 items) and friends’ support mean score (6-items) in four separate count models, and adjusted for SWA week, age, ethnicity, education, parity, body mass index and time since birth. We tested interactions between social support and ethnicity. Analyses were performed on complete cases and imputed data.

**Results:**

Based on imputed data, we observed that women who reported low and high support from family accumulated 16.2 (IQR: 6.1–39.1) and 18.6 (IQR: 5.0-46.5) MVPA minutes/day, respectively. Women who reported low and high support from friends accumulated 18.7 (IQR: 5.9–43.6) and 16.8 (IQR: 5.0-45.8) MVPA minutes/day. We observed a 12% increase in MVPA minutes/day for each additional increase in mean family support score (IRR = 1.12, 95% CI: 1.02 to 1.25). Women reporting high level of support from family on ‘discuss PA’, ‘co-participation’ and ‘take over chores’ accumulated 33%, 37% and 25% more MVPA minutes/day than women reporting low level of support respectively (‘discuss PA’: IRR = 1.33, 95% CI: 1.03 to 1.72, ‘co-participation’: IRR = 1.37, 95% CI: 1.13 to 1.66 and ‘take over chores’: IRR = 1.25, 95% CI: 1.02 to 1.54). Associations were not modified by ethnicity. No statistically significant association between support from friends and MVPA was observed. Similar results were found in complete case analyses, with a few exceptions.

**Conclusion:**

Overall family support and specific forms of support from family were associated with MVPA across ethnic groups, while support from friends was not associated with MVPA postpartum.

**Supplementary Information:**

The online version contains supplementary material available at 10.1186/s12889-023-15507-z.

## Background

There is a global public health challenge that low levels of physical activity (PA) exist in population sub-groups (i.e., ethnic groups, women) [1–3]. Insufficient levels of PA increase the risk for adverse health outcomes such as non-communicable diseases, depression, reduced quality of life and well-being [4–6]. Further, the prevalence of adverse health outcomes differs by ethnicity and sex [7]. For example, among minority ethnic groups in Western countries, women from South Asia were reported to have a higher risk of type 2 diabetes (27.5%) compared to Norwegians (2.9%) [7].

Factors that influence PA behavior are complex, and they have been organized in eight domains: health and health communication, political environment, social and cultural environment, psychosocial, institutional environment, physical environment and opportunity, social and material resources and migration context [8]. Factors across domains interplay, and the importance of factors may differ across the life-span [9].

Theory and empirical studies of social support suggest that the feeling of being cared for or supported promotes a sense of connection, self-esteem, control and self-efficacy that directly influence PA behaviors [10, 11]. Social support is defined by the *form* (received and perceived support), the *source* (i.e., family/friends) and the *function/type* (instrumental support, appraisal, informational, emotional and validation) [12]. Evidence suggests that social support (in the domain social and cultural environment) from caregivers is an important factor of PA behavior among children and adolescents manifested through encouragement and companionship [13]. Regarding adults, two systematic reviews of prospective studies (primarily Anglo-American) of the relationship between social support and self-reported PA (leisure time PA/moderate-to-vigorous PA) showed inconsistent results and depended on source and type of support [11, 12]. However, three studies based on objectively measured moderate-to-vigorous PA (MVPA) reported no association between social support and MVPA among adults [14–16]. There is a need to simultaneously assess multiple and specific types of social support to better understand the association with PA [12].

Transition periods such as postpartum influence maternal PA behavior [9]. Postpartum initiates incorporation of new daily routines to accommodate the newborn’s needs, and the need to cope with new family dynamics that may disrupt habitual PA [17]. We have previously reported that Western European women were more physically active than ethnic minority women in postpartum. The difference was 150–180 MVPA minutes /week more when estimated from bouts lasting at least 10 minutes [18]. Studies of social support’s role in postpartum PA are based on self-reported PA data indicating that both support from family and friends may facilitate PA [17, 19]. If the associations exist based on objective PA measures are unclear. An understanding of social support’s influence on postpartum PA across ethnic groups is required to inform public health initiatives and health care personnel as they are important sources of social support and provision of PA advice in postnatal care.

The aims were to examine (1) if overall social support for PA from family or friends were associated with objectively recorded MVPA postpartum, (2) if specific types of support from family or friends were associated with MVPA and (3) if associations differed across ethnic groups.

## Methods

### Design, population, setting and data collection

Data was obtained from the population-based STORK Groruddalen Cohort, described in detail elsewhere [20]. In short, pregnant women living in multi-ethnic city districts in Oslo, Norway, attending the Child Health Clinics for antenatal care, were recruited between May 2008 and May 2010, and followed from mean gestational week 15 to mean 14 weeks postpartum. Inclusion criteria were (1) living in one of the three city districts, (2) planning to give birth at one of the two study hospitals, (3) being in gestational week ≤ 20 at inclusion, (4) not having diseases necessitating intensive hospital follow-up during pregnancy, (5) not already included in the study with a pregnancy lasting ≥ 22 weeks, (6) able to communicate in Norwegian or any of the other eight languages, and (7) able to give informed consent [20]. Written informed consent was obtained from all participants. The Regional Committee for Medical and Health Research Ethics for South Eastern Norway (ref: 2007/894) and the Norwegian Data Inspectorate approved the study protocol [20]. Trained midwives collected questionnaires and clinical data at the Child health Clinics. All information material and questionnaires were translated to eight languages: Arabic, English, Sorani, Somali, Tamil, Turkish, Urdu, and Vietnamese, covering the largest ethnic groups in Oslo [20]. Professional translators assisted when needed. MVPA was objectively recorded on subsequent days after each visit. In this study we use data collected at visit 1 (mean gestational week 15) and the postpartum visit 3 (mean 14 weeks postpartum).

### Primary outcome

*Moderate- to vigorous physical activity* (MVPA) was objectively recorded with SenseWear Armband^™^ Pro 3 (SWA) (Body Media Inc, Pittsburg, PA, USA) at the postpartum visit. Women were asked to wear the SWA across the right triceps brachii 24 h per day over 4–7 successive days and remove it only for water activities. Raw data was integrated into 60-seconds epochs using the manufacturer’s software (SenseWear™ Professional Research Software Version 6.1, BodyMedia Inc.). The summed value of 1-minute epochs was used to estimate metabolic equivalents (METs). MVPA minutes were defined as minute epochs ≥ 3METs (1 MET = 3.5 ml O2 ·kg − 1 · min − 1) and minutes in MVPA bouts of ≥ 10 subsequent minutes were extracted with SQL Server Management Studio (Microsoft®) and SQL Server Express version 11.0.5058.0 (Microsoft®). A minimum of 2 valid monitoring days were required, where one valid day consisted of ≥ 19.2 h of SWA wear-time. As the women were still on parental leave we did not distinguish between weekdays or weekend days. SWA’s validity and reliability have been evaluated for healthy adults under free-living conditions against double-labelled water [21, 22].

### Social support

*Perceived social support* for PA was measured by a modified version of the Social Support for Exercise Scale at the postpartum visit on a 12-item scale with six items for support from family (in the household) and six items for support from friends (including family-members outside the household and acquaintances) [23]. Participants separately rated how often at the current time their family or friends had supported them to be physically active through specific types of support. Single items of support from family included: encourage PA, discuss PA, co-participation, take over chores, health benefits talk and share PA enjoyment. Single items of support from friends included: offered to do PA together, encourage PA, helpful reminders, co-participation, health benefits talk and share PA enjoyment. Responses were given on a 6-point Likert scale including “never”, “rarely”, “sometimes”, “often”, “very often” and “does not apply”. “Does not apply was recoded as “never” [23]. Women with missing data on any item were excluded from analyses. We used the mean score (range 1–5) for the six items on support from family (Cronbach’s alpha = 0.86), and the mean score (range 1–5) for the six items on support from friends (Cronbach’s alpha 0.87). Further, we analyzed each of the 12 social support items dichotomized into high (sometimes, often and very often) and low social support (never and rarely).

### Covariates and auxiliary variables

A direct acyclic graph (DAG) was drawn prior to analyses to depict causal structures of possible pathways and associations (Supplementary Fig. 1). Age, ethnicity, education, parity, and body mass index, kg/m^2^ (BMI) were identified as plausible confounders, all collected at visit 1, except for BMI where we considered data from postpartum (visit 3) to be more relevant. SWA weeks postpartum was calculated from offspring`s date of birth.

Ethnicity refers to the participant’s country of birth or her mother, if her mother was born outside Europe or North America [20] and was categorized as Western Europe, South Asia, Middle Eastern and other ethnicities (Eastern Europe, East Asia and Africa South of Sahara). Age was analyzed as a continuous variable. Parity was categorized as nulliparous and parous (≥ 1 children before the recent pregnancy). BMI kg/m^2^ was measured by Tanita-BC 418 MA (Tanita, Tokyo, Japan) and based on body height measured to the nearest 0.1 cm using a fixed stadiometer at inclusion. Participants’ highest level of education was categorized as primary school or less (≤ 10 years), high school (11–13 years) and university or college. Auxiliary variables were objectively recorded MVPA bouts (≥ 10 minutes) at visit 1 (recoded by SWA), the PA-level of the child’s father at visit 1, the participants’ self-reported control over being physically active and the participants’ frequency of slow walks per week. The child’s father’s PA-level was categorized as > 3 times/week, 1–3 times/week, 1–3 times/month, less often. Self-reported control over being physically active was measured on a 7-point scale (1 = totally agree, 7 = totally disagree) regarding 5 statements (Supplementary material 1) [24]. Habitual PA was measured by average duration per exercise of nine specific exercises lasting at least 10 minutes (Supplementary Table 1a). Slow walk was categorized by the frequency per week (never, 1 time/week, 2 times/week, 3–6 times per week and daily).

### Statistical methods

Participant’s characteristics are presented as mean (SD), frequencies and proportions as appropriate. Descriptive MVPA data are presented as median and inter quartile range (IQR). Women with complete data on all items for support from family or friends constituted the sample. We analyzed the association between social support (from family or friends) and MVPA in four separate models ; negative binomial (NB), zero-inflated NB, Poisson logit hurdle, and NB logit hurdle regression models. The NB model is used extensively in research to account for over-dispersed Poisson data [25]. Approximately 50% of the women did not accomplish any bouts of at least 10 consecutive minutes in MVPA or did not meet our criteria for valid activity days and were represented by zero counts in the data. The disparity between the observed and expected zero counts posed methodological challenges when we attempted to apply the Poisson and the NB models. To account for the excess zero counts in the data, we employed the zero-inflated [26] and hurdle regression models [27]. The major difference between the two models is in the way the zero counts are modelled. Therefore, we distinguished the excess zeros as either sampling zeroes; representing women who are able to accomplish bouts of at least 10 consecutive minutes in MVPA but did not achieve the number of minutes in the study period, or structural zeros; representing women who cannot accomplish bouts of at least 10 consecutive minutes in MVPA. Generally, the zero-inflated models are used to model data with excess structural and sampling zeros whereas the hurdle models assume that all zero counts are structural zeros [28]. The hurdle models applied consist of two components: a binary part coded (0/1) where a 0 denotes a woman who failed to accomplish bouts of at least 10 consecutive minutes in MVPA and a 1 represents a woman who successfully accomplished. Here, the hurdle is defined at zero minutes of MVPA. For women who crossed the hurdle, a truncated-at-zero count model (Poisson and NB) was used to model the positive counts (MVPA min/day > 0). For each count model, two separate adjusted models were fitted to the data. Model 1 was adjusted for postpartum SWA week, whereas model 2 was additionally adjusted for age, ethnicity, education, parity, and BMI. The fitted models were compared using Akaike information criteria (AIC) [29], where the model with the smallest AIC value was preferred (Supplementary Tables 2 and 3). The AIC for imputed data was computed in two steps: first by estimating the models for each imputed dataset to obtain an AIC estimate, and second by averaging AIC estimates over the entire set. Although the NB hurdle and the ZINB were indistinct able in some models, overall, the NB hurdle provided the best fit. We also checked for interactions between social support and ethnicity.

To account for potentially biased estimates due to missing MVPA data (n = 302, 47.6% of the sample), we performed multiple imputations [30] using the miceadds package in R [31]. Predictive mean matching was used to impute missing values and we generated 50 imputed data sets [32], which we exported to Stata for further analyses. The Stata command “mi estimate” was used to obtain pooled estimates across the 50 imputed datasets. We included variables from the analytic models and auxiliary variables in the dataset associated with MVPA level or missingness of MVPA (p < 0.05) [32, 33]. Values were also imputed for confounders and auxiliary variables with missing values. Missing analyses on sub-groups with and without missing data were conducted (Supplementary Tables 1a-c) [34]. We present model estimates obtained from the imputed data as our main results (Tables 4 and 5), and estimates from complete cases data in supplementary (Supplementary Tables 4 and 5). All statistical analyses were performed using software StataSE version 16.0 and the significance level was set at α = 0.05.

## Results

### Study sample and characteristics

Of the 823 women included at visit 1, 662 (80.4%) attended the postpartum visit and 636 had complete data on social support (Flow chart, Supplementary Fig. 2). Ethnic groups proportions were Western Europe (42.8%), South Asia (24.5%), Middle East (14.8%), and other ethnicities (17.9%) represented (Table 1). Of Western European women, 89% were ethnic Norwegian. Mean (SD) age, years of residence in Norway and BMI were 30.0 (4.8) years, 9.4 (7.8) years and 26.0 (4.9) kg/m^2^ respectively. A total of 53.7% did not have college/university education. The mean (SD) SWA monitoring week was postpartum week 13.7 (2.3).

Out of the total sample (Flow chart, Supplementary Figs. 2), 303 had incomplete data. Women with complete data were on average one year older, had lower BMI and more had higher educational level and were Western European, compared to women with incomplete data (Supplementary Table 1c), but did not differ substantially with respect to self-reported habitual PA and social support (Supplementary Tables 1a and 1b). The most common self-reported habitual PA were slow walk and brisk walk in both groups (Supplementary Table 1a). Reasons for missing SWA data (n = 302) were not accepting to wear SWA (n = 247) or having < 2 valid recorded PA days (n = 55).

**Table 1:** Characteristics of the cohort by ethnic groups. Values in mean (SD) or numbers (%)

	Postpartum^a^				
Characteristics	Total N = 636	Western Europe^b^ N = 272	South Asia N = 156	Middle East N = 94	Other ethnicities N = 114
*Inclusion*, *mean gestational week 15*					
Age, years (mean/SD)	30.0 (4.8)	31.1 (4.3)	29.0 (4.3)	29.6 (5.5)	29.6 (5.2)
BMI, kg/m^2^ (mean/SD)^d^	26.0 (4.9)	25.7 (4.9)	25.6 (4.2)	27.7 (5.3)	26.1 (5.3)
Marital status, n (%)					
Single	22 (3.5)	5 (1.8)	1 (0.6)	3 (3.2)	13 (11.4)
Co-habitant	613 (96.5)	267 (98.2)	155 (99.4)	90 (96.8)	101 (88.6)
Parity, n (%)					
Nulliparous	294 (46.2)	140 (51.5)	66 (42.3)	32 (34.0)	56 (49.1)
Parous	342 (53.8)	132 (48.5)	90 (57.7)	62 (66.0)	58 (50.9)
Education, n (%)					
Primary school or less	94 (14.8)	5 (1.9)	32 (20.5)	32 (34.0)	25 (22.1)
High school / secondary	246 (38.9)	81 (30.0)	74 (47.4)	45 (47.9)	46 (40.7)
College / University	293 (46.3)	184 (68.1)	50 (32.1)	17 (18.1)	42 (37.2)
*14 weeks postpartum*					
SenseWear Armband week, mean (SD)^d^	13.7 (2.3)	13.5 (2.1)	13.7 (2.3)	14.0 (2.6)	13.8 (2.3)
Valid MVPA data^c^, n (%)	334 (53)	171 (63)	67 (43)	41 (44)	55 (48)

SWA: SenseWear Armband

BMI: Body mass index

^a^ Postpartum = mean 14 weeks postpartum

^b^ Western Europe includes 89% women with ethnic Norwegian background

^c^Valid MVPA data = at least 2 days with 19.2 h of SWA wear time

^d^Missing (total sample); BMI (n = 4), marital status (n = 1), education (n = 3), SenseWear Armband week, valid MVPA data (n = 302)

Other ethnicities: Eastern Europe, East Asia and Africa South of Sahara

### Social support and MVPA patterns

Mean (SD) family support score was 2.9 (1.0), while mean (SD) friends’ support score was 2.5 (0.9) (Table 2). On four of six single items for support from family (encourage PA, discuss PA, health benefits talk, and share PA enjoyment) > 70% reported a high level of support. A lower proportion of women reported high level of support on the single items ‘take over chores’ (48%) and ‘co-participation’ (42%).

High support from friends was reported among 55 to 61% women on four of six items (offered to do PA together, discuss PA, health benefits talk and share PA enjoyment). A lower proportion reported high support from friends on the items ‘helpful reminders’ and ‘co-participation.’ Across all items a smaller proportion of women with Middle Eastern background reported high support from friends compared to other ethnic groups, except the item ‘health benefits talk’.

**Table 2:** Distribution of support from family and friends by ethnic groups postpartum

		Total n = 636	Western Europe n = 272	South Asia n = 156	Middle East n = 94	Other ethnicities n = 114
		n (%)	n (%)	n (%)	n (%)	n (%)
**Family support**						
Overall family support	low	156 (24.5)	70 (25.7)	31 (19.9)	22 (23.4)	33 (29.0)
	high	480 (75.5)	202 (74.3)	125 (80.1)	72 (76.6)	81 (71.0)
Encourage PA	low	177 (27.8)	70 (25.7)	45 (28.9)	31 (33.0)	31 (27.2)
	high	459 (72.2)	202 (74.3)	111 (71.1)	63 (67.0)	83 (72.8)
Discuss PA	low	180 (28.3)	66 (24.3)	45 (28.9)	27 (28.7)	42 (36.8)
	high	456 (71.7)	206 (75.7)	111 (71.1)	67 (71.3)	72 (63.2)
Co-participation	low	372 (58.5)	164 (60.3)	88 (56.4)	60 (63.8)	60 (52.6)
	high	264 (41.5)	108 (39.7)	68 (43.6)	34 (36.2)	54 (47.4)
Take over chores	low	331 (52.0)	138 (50.7)	84 (53.9)	52 (55.3)	57 (50.0)
	high	305 (48.0)	134 (49.3)	72 (46.1)	42 (44.7)	57 (50.0)
Health benefits talk	low	187 (29.4)	104 (38.2)	30 (19.2)	24 (25.5)	29 (25.4)
	high	449 (70.6)	168 (61.8)	126 (80.8)	70 (74.5)	85 (74.6)
Share PA enjoyment	low	192 (30.2)	85 (31.3)	40 (25.6)	28 (29.8)	39 (34.2)
	high	444 (69.8)	187 (68.8)	116 (74.4)	66 (79.2)	75 (65.8)
**Friends’ support**						
Overall friends’ support	low	232 (36.5)	114(41.9)	46 (29.5)	38 (40.4)	34 (29.8)
	high	404 (63.5)	158 (58.1)	110 (70.5)	56 (59.6)	80 (70.2)
Offered to do PA together	low	287 (45.1)	108 (39.7)	71 (45.5)	52 (55.3)	56 (49.1)
	high	349 (54.9)	164 (60.3)	85 (54.5)	42 (44.7)	58 (50.9)
Encourage PA	low	282 (44.3)	127 (46.7)	58 (37.2)	48 (51.1)	49 (43.0)
	high	354 (55.7)	145 (53.3)	98 (62.8)	46 (48.9)	65 (57.0)
Helpful reminders	low	372 (58.5)	164 (60.3)	84 (53.9)	64 (68.1)	60 (52.6)
	high	264 (41.5)	108 (39.7)	72 (46.1)	30 (31.9)	54 (47.4)
Co-participation	low	456 (71.7)	203 (74.6)	107 (68.6)	71 (75.5)	75 (65.8)
	high	180 (28.3)	69 (25.4)	49 (31.4)	23 (24.5)	39 (34.2)
Health benefits talk	low	277 (43.5)	159 (58.5)	45 (28.9)	44 (46.8)	29 (25.4)
	high	359 (56.5)	113 (41.5)	111 (71.1)	50 (53.2)	85 (74.6)
Share PA enjoyment	low	249 (39.2)	113 (41.5)	53 (34.0)	43 (45.7)	40 (35.1)
	high	387 (60.8)	159 (58.5)	103 (66.0)	51 (54.3)	74 (64.9)

PA: physical activity

Median (IQR) MVPA was higher in women who reported high support from family compared with those reporting low support 20.8 (5.0-46.5) versus15.5 (5.0-43.5) min/day, (Table 3). Median (IQR) MVPA was higher in women who reported low support from friends compared with those reporting high support 18.7 (5.9–43.6) versus 15.5 (4.6–46.5) min/day. Across all measures of support from family or friends (mean score and single items), Western European women accumulated more MVPA min/day than women in other ethnic groups, regardless of the level of social support.

**Table 3:** Median MVPA minutes/day with 25th -75th percentile by level of family and friends’ support

		Moderate-to-vigorous physical activity minutes/day postpartum									
		Total N = 334		Western Europe N = 171		South Asia N = 67		Middle East N = 41		Other ethnicities N = 55	
		n	Median (IQR)	n	Median (IQR)	n	Median (IQR)	n	Median (IQR)	n	Median (IQR)
**Family support**											
Overall support	low	80	16.2 (6.1–39.1)	44	23.5 (10.0-43.9)	11	10.8 (0.0-27.7)	9	7 (0.0–13.0)	16	17.3 (5.3–52.2)
	high	254	18.6 (5.0-46.5)	127	31.0 (12.8–64.5)	56	6.4 (0.0-24.7)	32	7.7 (1.3–31.5)	39	12.0 (3.7–19.0)
Encourage PA	low	89	15.3 (5.0–41.0)	44	18.3 (11.0-55.9)	18	11.7 (3.5–28.5)	9	5.0 (0.0-16.5)	18	8.5 (3.7–22.5)
	high	245	20 (5.5–46.5)	127	32.5 (12.6–62.5)	49	6 (0.0–23.0)	32	7.7 (1.3–24.3)	37	13.3 (5.0–35.0)
Discuss PA	low	92	13.1 (4.1–32.3)	42	23.5 (9.0-57.3)	17	5.0 (0.0-16.3)	10	6.0 (0.0-8.7)	23	12.3 (3.8–35.0)
	high	242	20.8 (5.8–48.0)	129	28.7 (12.7–62.5)	50	7.8 (0.0-30.5)	31	11.7 (0.0-43.7)	32	11.9 (4.3–29.9)
Co-participation	low	188	14.0 (3.7–37.0)	102	19.5 (9.0-45.8)	37	6.0 (0.0-20.8)	23	5.0 (0.0-19.3)	26	10.5 (3.5–41.0)
	high	146	23.2 (6.5–56.7)	69	46.5 (23.0–73.0)	30	7.2 (0.0–31.0)	18	16.4 (3.7–50.0)	29	13.5 (5.0-19.3)
Take over chores	low	169	15.2 (4.0 -39.8)	85	26.5 (9.7–52.7)	36	5.0 (0.0-17.8)	21	5.0 (0.0–13.0)	27	11.8 (3.7–41.0)
	high	165	21.8 (6.8–50)	86	31.1 (12.8–69.5)	31	15.0 (0.0-31.3)	20	20.6 (1.7–40.3)	28	12.8 (4.8–19.1)
Health benefits talk	low	101	22.8 (8.4–61.3)	65	34.8 (13.2–76.5)	12	5.5 (1.8–22.3)	8	10.7 (6.0-33.3)	16	20.8 (7.4–42.3)
	high	233	16.7 (4.6–40.8)	106	26.8 (11.0-52.7)	55	7.5 (0.0-26.3)	33	5.8 (0.0-22.5)	39	11.8 (3.7–17.8)
Share PA enjoyment	low	93	18.3 (7.0-39.8)	53	25.0 (12.6–48.0)	13	10.8 (4.0–17.0)	7	2.5 (0.0-16.5)	20	17.3 (7.4–38.0)
	high	241	17.7 (4.7–46.5)	118	32.9 (12.0-62.5)	54	6.4 (0.0-26.3)	34	8.5 (3.0–26.0)	35	11.0 (3.7–17.8)
**Friends’ support**											
Overall support	low	176	18.7 (5.9–43.6)	104	26.0 (11.2–55.0)	26	9.4 (0.0-26.3)	21	7.0 (0.0–26.0)	25	12.0 (3.5–42.0)
	high	158	15.5 (4.6–46.5)	67	34.8 (12.6–69.0)	41	5.0 (0.0–23.0)	20	7.6 (2.8–20.7)	30	12.2 (5.0-19.3)
Offered to do PA together	low	137	15.3 (3.5–39.8)	69	25.0 (9.0–55.0)	23	5.0 (0.0–23.0)	20	5.4 (0.0-29.3)	25	14.0 (3.5–22.5)
	high	197	20.0 (6.8–48.4)	102	33.6 (13.2–62.5)	44	9.6 (3.0-27.4)	21	11.7 (3.0-22.5)	30	12.0 (5.0–42.0)
Encourage PA	low	147	18.3 (5.8–50.0)	85	27.8 (12.0-61.3)	21	8.0 (3.3–26.3)	18	6.0 (0.0–37.0)	23	11.8 (2.5–22.5)
	high	187	16.7 (5.0-43.3)	86	27.5 (12.5–61.3)	46	5.5 (0.0–23.0)	23	8.4 (3.0-19.7)	32	12.8 (5.8–38.0)
Helpful reminders	low	197	19.3 (5.0–48.0)	106	28.3 (12.7–56.7)	37	6.8 (0.0–23.0)	28	8.5 (0.0-40.3)	26	11.9 (3.5–42.3)
	high	137	15.5 (5.3–41.0)	65	25.0 (11.5–62.5)	30	6.8 (0.0–31.0)	13	5.8 (2.5–13.0)	29	13.3 (6.5–19.3)
Co-participation	low	232	16.6 (5.0-43.5)	127	26.0 (10.5–55.0)	41	6.8 (0.0-23-0)	29	5.0 (0.0-21.5)	35	10.5 (3.7–42.0)
	high	102	21.0 (6.8–48.5)	44	41.7 (20.6–80.8)	26	8.8 (3.5–28.5)	12	15.7 (4.7–24.3)	20	13.7 (7.0-30.3)
Health benefits talk	low	151	22.8 (7.3–48.0)	104	27.9 (13.1–57.0)	16	9.4 (5.5–27.0)	15	11.7 (0.0–37.0)	16	4.4 (0.0-33.8)
	high	183	13.5 (4.0-43.7)	67	26.5 (9.0-65.4)	51	4.4 (0.0–23.0)	26	6.9 (3.0-19.7)	39	12.3 (6.5–35.0)
Share PA enjoyment	low	115	18.3 (6.0–38.0)	64	23.5 (11.2–42.8)	18	6.4 (0.0-21.8)	14	20.4 (5.0–37.0)	19	15.0 (2.5–46.5)
	high	219	17.0 (4.0-49.7)	107	37.0 (12.5–69.0)	49	7.5 (0.0-28.5)	27	5.8 (0.0-19.7)	36	11.5 (4.8–22.5)

MVPA: moderate-to-vigorous physical activity

IQR: interquartile range in average minutes/day

PA: physical activity

Other ethnicities: Eastern Europe, East Asia and Africa South of Sahara

### Associations between support from family and MVPA

#### The binary part of hurdle NB

The odds ratio in the binary part of the NB-logit hurdle of imputed data in Table 4 shows that none of the family variables were significantly associated with the odds of a women not accomplishing bouts of at least 10 consecutive minutes in MVPA.

#### The count part of hurdle NB

For a given family support item, an IRR estimate > 1 indicates an increase in MVPA min/day whereas an IRR estimate < 1 indicates a decrease in MVPA min/day.

In the adjusted hurdle NB of imputed data (Table 4, model 2), our results showed that a 1-point increase in overall mean family support score (scale 1–5) was significantly associated with a 12% increase in MVPA min/day. Further, we observed significant increases in counts of MVPA min/day of 33%, 37% and 25% among women who reported high support from family on the items ‘discuss PA’, ‘co-participation’ and ‘take over chores’ than those who did not respectively. In analysis of complete cases a significant interaction was observed between ethnicity and the item ‘co-participation’ (Supplementary Fig. 3). Western European women who reported high support manifested through ‘co-participation’ had 50% more MVPA min/day than those who did not. Overall, we found agreements between results from imputed data and complete case analyses (Tables 4 and Supplementary Table 2).

**Table 4:** Associations between family support and MVPA min/day based on imputed data

Family support		NB	Zero inflated NB (ZINB)	Hurdle Poisson	Hurdle NB
**Count part**	Model	IRR (95% CI)	IRR (95% CI)	IRR (95% CI)	IRR (95% CI)
Overall support	1	1.11 (0.98, 1.26)	1.09 (0.98, 1.22)	1.09 (1.02, 1.16) ^***^	1.09 (0.98, 1.22)
	2	1.14 (1.01, 1.31) ^*^	1.12 (1.01, 1.25) ^*^	1.10 (1.03, 1.18) ^**^	1.12 (1.01, 1.25) ^*^
Encourage PA	1	1.10 (0.83, 1.46)	1.09 (0.86, 1.38)	1.09 (0.95, 1.25)	1.09 (0.86, 1.38)
	2	1.11 (0.82, 1.50)	1.11 (0.87, 1.40)	1.05 (0.91, 1.21)	1.11 (0.87, 1.40)
Discuss PA	1	1.44 (1.07, 1.95) ^*^	1.39 (1.08, 1.79) ^**^	1.38 (1.17, 1.63) ^***^	1.39 (1.08, 1.79) ^*^
	2	1.38 (1.01, 1.88) ^*^	1.33 (1.03, 1.72) ^*^	1.25 (1.05, 1.49) ^**^	1.33 (1.03, 1.72) ^*^
Co-participation	1	1.42 (1.12, 1.80) ^**^	1.33 (1.09, 1.62) ^**^	1.31 (1.19, 1.45) ^***^	1.33 (1.09, 1.62) ^**^
	2	1.46 (1.15, 1.87) ^**^	1.37 (1.13, 1.66) ^**^	1.30 (1.17, 1.45) ^***^	1.37 (1.13, 1.66) ^**^
Take over chores	1	1.28 (1.00, 1.64) ^*^	1.24 (1.01, 1.53) ^*^	1.23 (1.09, 1.39) ^**^	1.24 (1.01, 1.53) ^*^
	2	1.30 (1.00, 1.68) ^*^	1.25 (1.02, 1.53) ^*^	1.21 (1.07, 1.37) ^**^	1.25 (1.02, 1.54) ^*^
Health benefits talk	1	0.70 (0.54, 0.91) ^**^	0.73 (0.59, 0.91) ^**^	0.74 (0.66, 0.83) ^**^	0.73 (0.59, 0.91) ^*^
	2	0.86 (0.66, 1.13)	0.87 (0.70, 1.08)	0.84 (0.75, 0.95) ^**^	0.87 (0.70, 1.08)
Share PA enjoyment	1	1.16 (0.90, 1.50)	1.21 (0.97, 1.50)	1.21 (1.08, 1.35) ^**^	1.21 (0.97, 1.50)
	2	1.16 (0.89, 1.49)	1.19 (0.97, 1.46)	1.17 (1.05, 1.30) ^**^	1.19 (0.97, 1.46)
**Binary part**				OR (95% CI)	OR (95% CI)
Overall support	1			0.91 (0.69, 1.21)	0.91 (0.69, 1.21)
	2			0.87 (0.64, 1.18)	0.87 (0.64, 1.18)
Encourage PA	1			0.94 (0.50, 1.79)	0.94 (0.50, 1.79)
	2			1.02 (0.51, 2.03)	1.02 (0.51, 2.03)
Discuss PA	1			0.75 (0.43, 1.33)	0.75 (0.43, 1.33)
	2			0.81 (0.43, 1.51)	0.81 (0.43, 1.51
Co-participation	1			0.64 (0.37, 1.10)	0.64 (0.37, 1.10)
	2			0.63 (0.35, 1.14)	0.63 (0.35, 1.14)
Take over chores	1			0.81 (0.47, 1.40)	0.81 (0.47, 1.40)
	2			0.80 (0.44, 1.46)	0.80 (0.44, 1.46)
Health benefits talk	1			1.45 (0.78, 2.67)	1.45 (0.78, 2.67)
	2			1.17 (0.60, 2.27)	1.17 (0.60, 2.27)
Share PA enjoyment	1			1.24 (0.67, 2.29)	1.24 (0.67, 2.29)
	2			1.23 (0.63, 2.42)	1.23 (0.63, 2.42)

Statistically significant results are indicated with an asterisk (*). ^*^P < 0.05, ^**^P < 0.01, ^***^P < 0.001. Based on the AIC given in Supplementary Tables 2, model 2 of the NB-logit hurdle fitted the data best

PA: physical activity

MVPA: moderate- to vigorous physical activity

Model 1: adjusted for SenseWear Armband week

Model 2: adjusted for SenseWear Armband, ethnicity, age, education, parity, and body mass index

### Associations between support from friends and MVPA

#### The binary part of hurdle NB

The odds ratio in the binary part of the NB-logit hurdle of imputed data in Table 5 show that none of the friends’ variables were significantly associated with the odds of a women not accomplishing bouts of at least 10 consecutive minutes in MVPA.

#### The count part of hurdle NB

We found no significant associations between support from friends and MVPA in imputed data (Table 5). However, in the complete case analyses, women who reported high support from friends on the item ‘co-participation’ had a 29% significant increase in MVPA min/day than those who did not (Supplementary Table 3). Further, the odds of a women failing to accomplish bouts of at least 10 consecutive minutes in MVPA decreased by 58% (OR = 0.42, 95% CI: 0.22 to 0.80) among women whose friends had offered to do PA with them based on complete case analysis (Supplementary Table 3). Our findings also showed significant interactions between the items ‘co-participation’ and ‘share PA enjoyment’ and ethnicity in complete case analyses (Supplementary Fig. 3). The interactions suggested that Western European women who reported high support manifested through ‘co-participation’ and ‘share PA enjoyment’ had an average of 40% and 30% more MVPA min/day than women with low support, respectively (Supplementary Fig. 3). No further associations were found in the complete case analyses.

**Table 5:** Associations between friends’ support and MVPA min/day based on imputed data

Friends’ support		NB	Zero-inflated NB	Hurdle Poisson	Hurdle NB
**Count part**	Model	IRR (95% CI)	IRR (95% CI)	IRR (95% CI)	IRR (95% CI)
Overall support	1	1.04 (0.90, 1.19)	1.01 (0.90, 1.14)	1.01 (0.95, 1.07)	1.01 (0.90, 1.14)
	2	1.09 (0.95, 1.26)	1.06 (0.95, 1.19)	1.05 (0.98, 1.12)	1.06 (0.95, 1.19)
Offered to do PA together	1	1.24 (0.97, 1.59)	1.13 (0.98, 1.41)	1.12 (0.99, 1.28)	1.13 (0.91, 1.41)
	2	1.22 (0.96, 1.56)	1.13 (0.92, 1.38)	1.10 (0.97, 1.24)	1.13 (0.92, 1.38)
Encourage PA	1	0.98 (0.76, 1.25)	0.96 (0.78, 1.18)	0.96 (0.86, 1.08)	0.96 (0.78, 1.18)
	2	1.04 (0.81, 1.35)	1.02 (0.83, 1.24)	0.99 (0.88, 1.11)	1.02 (0.83, 1.24)
Helpful reminders	1	0.96 (0.76, 1.22)	0.95 (0.78, 1.16)	0.95 (0.87, 1.05)	0.95 (0.78, 1.16)
	2	0.96 (0.76, 1.21)	0.95 (0.79, 1.14)	0.96 (0.87, 1.05)	0.95 (0.79, 1.14)
Co-participation	1	1.24 (0.93, 1.65)	1.17 (0.92, 1.49)	1.17 (1.00, 1.35) ^*^	1.17 (0.92, 1.49)
	2	1.29 (0.96, 1.73)	1.22 (0.96, 1.54)	1.21 (1.04, 1.41) ^*^	1.22 (0.96, 1.54)
Health benefits talk	1	0.82 (0.65, 1.05)	0.84 (0.68, 1.03)	0.84 (0.75, 0.94) ^**^	0.84 (0.68, 1.03)
	2	0.95 (0.72, 1.24)	0.94 (0.76, 1.17)	0.94 (0.83, 1.06)	0.94 (0.76, 1.17)
Share PA enjoyment	1	1.15 (0.89, 1.49)	1.16 (0.93, 1.45)	1.15 (1.01, 1.31) ^*^	1.16 (0.93, 1.45)
	2	1.14 (0.88, 1.46)	1.16 (0.95, 1.41)	1.16 (1.02, 1.31) ^*^	1.16 (0.95, 1.41)
**Binary part**				OR (95% CI)	OR (95% CI)
Overall support	1			0.88 (0.66, 1.17)	0.88 (0.66, 1.17)
	2			0.83 (0.61, 1.13)	0.83 (0.61, 1.13)
Offered to do PA together	1			0.58 (0.33, 1.01)	0.58 (0.33, 1.01)
	2			0.63 (0.35, 1.14)	0.63 (0.35, 1.14)
Encourage PA	1			0.93 (0.53, 1.61)	0.93 (0.53, 1.61)
	2			0.87 (0.48, 1.58)	0.87 (0.48, 1.58)
Helpful reminders	1			0.94 (0.55, 1.58)	0.94 (0.55, 1.58)
	2			0.94 (0.53, 1.65)	0.94 (0.53, 1.65)
Co-participation	1			0.69 (0.38, 1.26)	0.69 (0.38, 1.26)
	2			0.65 (0.34, 1.24)	0.65 (0.34, 1.24)
Health benefits talk	1			1.16 (0.68, 1.98)	1.16 (0.68, 1.98)
	2			0.99 (0.55, 1.77)	0.99 (0.55, 1.77)
Share PA enjoyment	1			1.05 (0.61, 1.80)	1.05 (0.61, 1.80)
	2			1.09 (0.60, 1.97)	1.09 (0.60, 1.97)

Statistically significant results are indicated with an asterisk (*). ^*^P < 0.05, ^**^P < 0.01, ^***^P < 0.0001. Based on the AIC given in Supplementary Tables 3, model 2 of the NB-logit hurdle fitted the data best

PA: physical activity

MVPA: moderate- to vigorous physical activity

Model 1: adjusted for SenseWear Armband week

Model 2: adjusted for SenseWear Armband, ethnicity, age, education, parity, and body mass index

## Discussion

To the best of our knowledge, this is the first population-based study investigating associations between PA support from family and friends and objectively recorded MVPA in a multi-ethnic population of postpartum women, using multiple measures of social support. We found that family support mean score was associated with MVPA, and that, women who perceived high levels of support from family in terms of discussion about PA, co-participation in PA and take over chores had more MVPA regardless of ethnicity. However, we found no association between support from friends and MVPA. Thus, sources of social support and types of support were differently associated with MVPA.

### Methods and design - strengths and limitations

The use of objectively recorded MVPA strengthens the validity of the MVPA estimates as recall and social desirability biases are reduced [35]. Our restriction of MVPA to bouts of ≥ 10 min “filters out” sporadic MVPA minutes (e.g., walking up the stairs), while MVPA accumulated during sustained activities such as walking, jogging, aerobic and strength training is more likely to be “filtered in”. It is therefore plausible that MVPA reflected behaviors that are responsive to social support. The Social Support Exercise Scale survey has good reliability and criterion validity among mothers aged 45 years or younger [23], and is the most frequently used measure of social support [12]. The questionnaire includes items representing distinct types and sources of support, and the items are PA-specific which is reported to be more sufficient than non-specific in attempt to target PA behavior and reflects that distinct types of social support may be differently associated with MVPA, in contrast to sum scores [36]. The combination of objectively recorded MVPA data and criterion validated social support strengthens the internal validity of the analyses. The use of translated study material and questionnaires, and use of interpreters during the data collection, were key factors in recruiting a representative multi-ethnic sample also including women with poor Norwegian language skills [20]. To reduce the potential selection bias from the large proportion of missing MVPA data, we used multiple imputation, and report results from complete cases and imputed data for transparency. There are also weaknesses to report. To avoid further reductions in sample size, we used MVPA data with at least two valid SWA days, even though three to five days are recommended to estimate habitual physical activity [37]. The cross-sectional design precludes interpretation in terms of causal relationships.

Even though the overall missingness for the response variable was almost 50%, the distribution of social support from family or friends and self-reported habitual PA were comparable between women with and without complete data. However, the observed variation in age, BMI, educational level and especially the large proportion of western European women in complete cases might explain the discrepancy in results from complete cases and imputed data. Further, missing in PA data can be influenced by cultural factors such as social acceptance for PA, ideal behavior, and ‘lack of exercise’ culture among ethnic minorities [38]. Hence, we consider the imputed analyses to be less biased and results from imputed data are further discussed.

Our MVPA data exhibited problems of over-dispersion where the variance far exceeded the distributional mean of MVPA due to an excess count of zero minutes of MVPA per day. Although the NB model is preferred over the Poisson model when the over-dispersion is due to unobserved heterogeneity, the zero-inflated and hurdle (Poisson and NB) are preferred when the over-dispersion is due to excess zeroes in the data. Our results showed that the zero-inflated and hurdle NB were more flexible in accounting for the excess zeroes and provided better fit of the data than the standard NB model and the hurdle Poisson model. However, our zero-inflated and hurdle NB models were indistinguishable in terms of model estimates and goodness of fit, although the hurdle NB was slightly better in some models based on the AIC statistic.

### Associations between support from family and MVPA

The results shows that family support may promote PA in postpartum women. One-point increase in score for overall family support increased accumulated MVPA min/day by 12%, while women with high family support manifested through PA discussions, co-participation and take over chores accumulated between 25-37% more MVPA min/day than women with low support. Such differences in accumulated MVPA min/day could be clinically important [39]. Evidence from quantitative and qualitative studies of self-reported PA postpartum supports our findings that overall support from family might increase engagement in PA [17, 19]. Specifically, in line with our results, postpartum women reported that the most important source and types of support to influence PA and moderate PA-intensity were family [40–44] and support manifested through co-participation [19] and emotional support (e.g., women’s perception that PA is desirable to their partner) [17]. Thus, our findings show the same trend as previous research and strengthen the belief in an association between family support and MVPA.

According to theories of social support and PA behavior, social support for PA is seen as a factor that may increase i.e., self-efficacy and thus confidence to perform the desirable behavior [45]. This, could explain higher levels of PA, despite the usual barriers of childcare, time availability and competing priorities among postpartum women [17]. Further, family members who co-participate in PA may provide assistance with childcare promoting higher PA intensity while they do PA together [46]. Another mechanism can be the perceived social pressure to perform PA (social norms) [47] leading to increased PA, facilitated through family discussions about PA and PA co-participation. However, previous studies on social norms showed no impact on MVPA [11]. Further, family members who are physically active might be more likely to co-participate and discuss PA, which may reduce the perceived barriers to PA and increase postpartum PA engagement, compared to the influence of inactive family members [48].

Thus, overall family support, particularly co-participation, discussion about PA and take over chores seem to help women overcome PA barriers through confidence and perceived social desirability to engage in PA in the stage and context about 14 weeks postpartum. Further, the observed associations between support from family and PA was found across socio-economic and ethnically diverse population, which are consistent with findings based on self-reported PA from postpartum [17].

### Associations between support from friends and MVPA

In contrast to systematic reviews from quantitative and qualitative studies [17, 19], we did not find associations between support from friends’ and MVPA. The discrepancy may result from methodological differences, especially the use of self-reported PA including domains of PA versus objectively measured PA considered more valid, different measures of social support and postpartum stage [35]. Findings from qualitative studies suggest that postpartum women perceive support from friends as less crucial compared to support from family [19]. The lesser importance of support from friends may be due to the postpartum context and stage, as social contact with friends is reduced [11], and the role of friends may therefore be less important for PA behavior than family support [40, 42, 49]. The reduced contact with friends in the postpartum period may explain why no association was observed in the current study. Postpartum women may be less inclined to socialize with their friends as responsibilities and care for the newborn baby might draw the attention toward the family and underline the need of support from family in postpartum [17].

A political factor that challenges comparability of PA estimates in postpartum across countries, is that maternal leave diverges across countries [50]. Thus, women’s opportunities to prioritize PA will differ across countries. In Norway employed women giving birth to a child have the right to paid parental leave from birth until the child is around one year old, which needs to be taken into account.

### Implications

The current study highlights that postpartum health may be enhanced through increased PA facilitated by overall support from family and family members who discuss PA, co-participate in PA and take over chores. Further, the findings of no interactions indicated that the effect on level of PA is irrespective of ethnicity in our study. Thus, inactive postpartum women may increase PA level if provided support from family. Nevertheless, trials designed to test the effect of a family-oriented approach in postpartum care on MVPA are needed to determine causal associations that can enable the primary health care sector to develop targeted and dynamic interventions to reduce social inequalities in health among postpartum women. Further, the importance of support from family may vary at different postpartum stages. Hence, more research is needed at later postpartum periods with objectively recorded PA data.

## Conclusions

Our findings support the importance of overall support from family for MVPA level and that specific types of support for PA behavior appear to have a larger impact. In particular, family members who discuss PA, co-participate and take over chores had an impact on postpartum women. Further, the associations did not differ across ethnic groups. Support from friends seems less important for MVPA postpartum.

## Electronic supplementary material

Below is the link to the electronic supplementary material.

Supplementary Material 1

Supplementary Material 2

Supplementary Material 3

Supplementary Material 4

Supplementary Material 5

Supplementary Material 6

Supplementary Material 7

Supplementary Material 8

Supplementary Material 9

Supplementary Material 10

Supplementary Material 11

Supplementary Material 12

## Data Availability

Due to ethical restrictions and patient confidentiality, not all data can be made publicly available. Data are available upon request from the Medical Faculty at the University of Oslo for researchers who meet the criteria for access to confidential data. Access can be arranged by direct request to co-author Anne Karen Jenum (a.k.jenum@medisin.uio.no).

## Abbreviations

MVPA: moderate-to-vigorous physical activity
PA: physical activity
SD: standard deviation
95% Cl: 95% confidence interval
SWA: SenseWear™ Armband
