# Supplementary figures and images for: Associations between social support and physical activity in postpartum: a Norwegian multi-ethnic cohort study
Source: BMC Public Health. 2023 Apr 17;23:702. doi: 10.1186/s12889-023-15507-z (PMC10111809; doi:10.1186/s12889-023-15507-z)

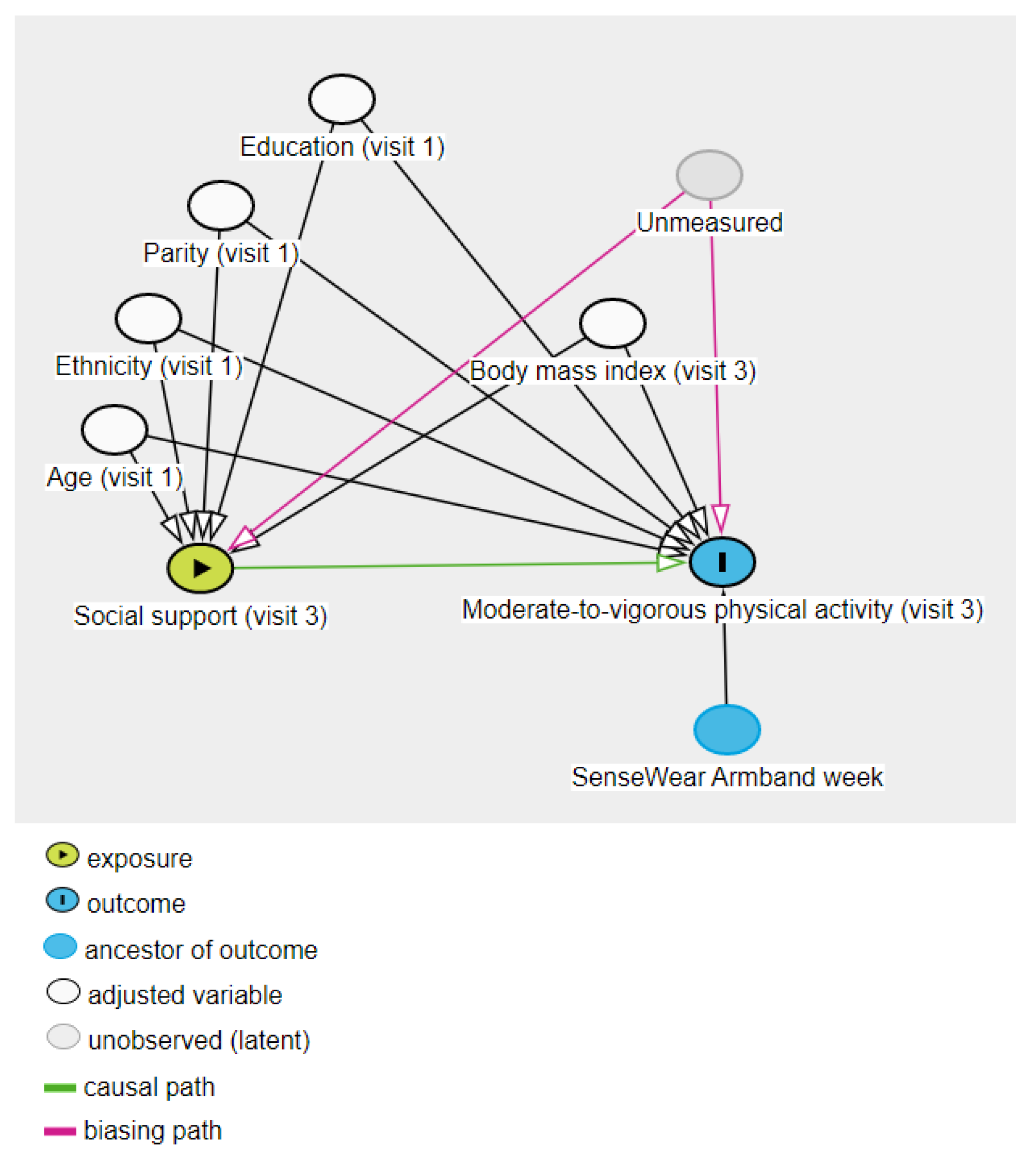

Supplement: Supplementary file 1 — Supplementary Material 1 [file 12889_2023_15507_MOESM1_ESM.tif]

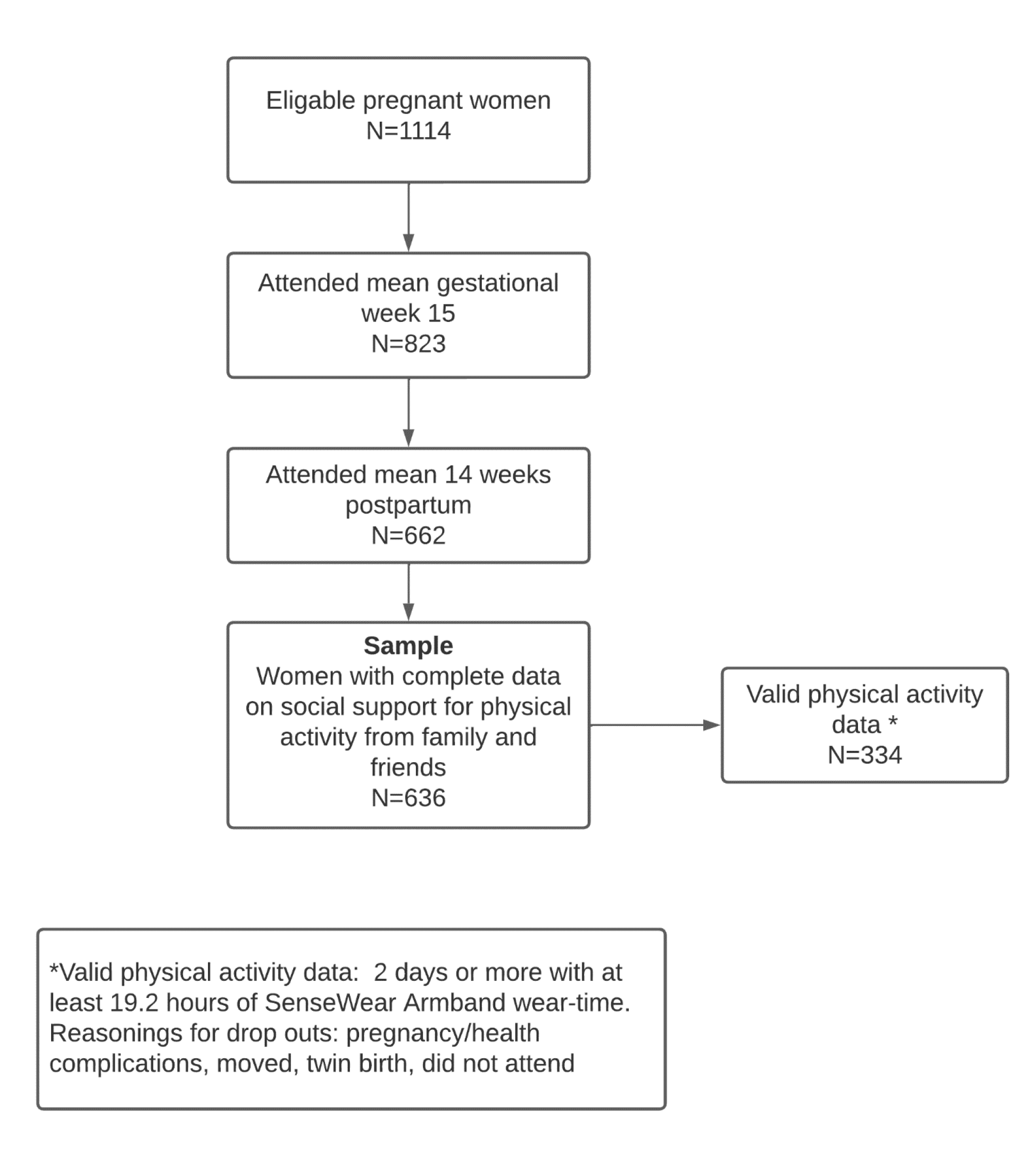

Supplement: Supplementary file 10 — Supplementary Material 10 [file 12889_2023_15507_MOESM10_ESM.tif]

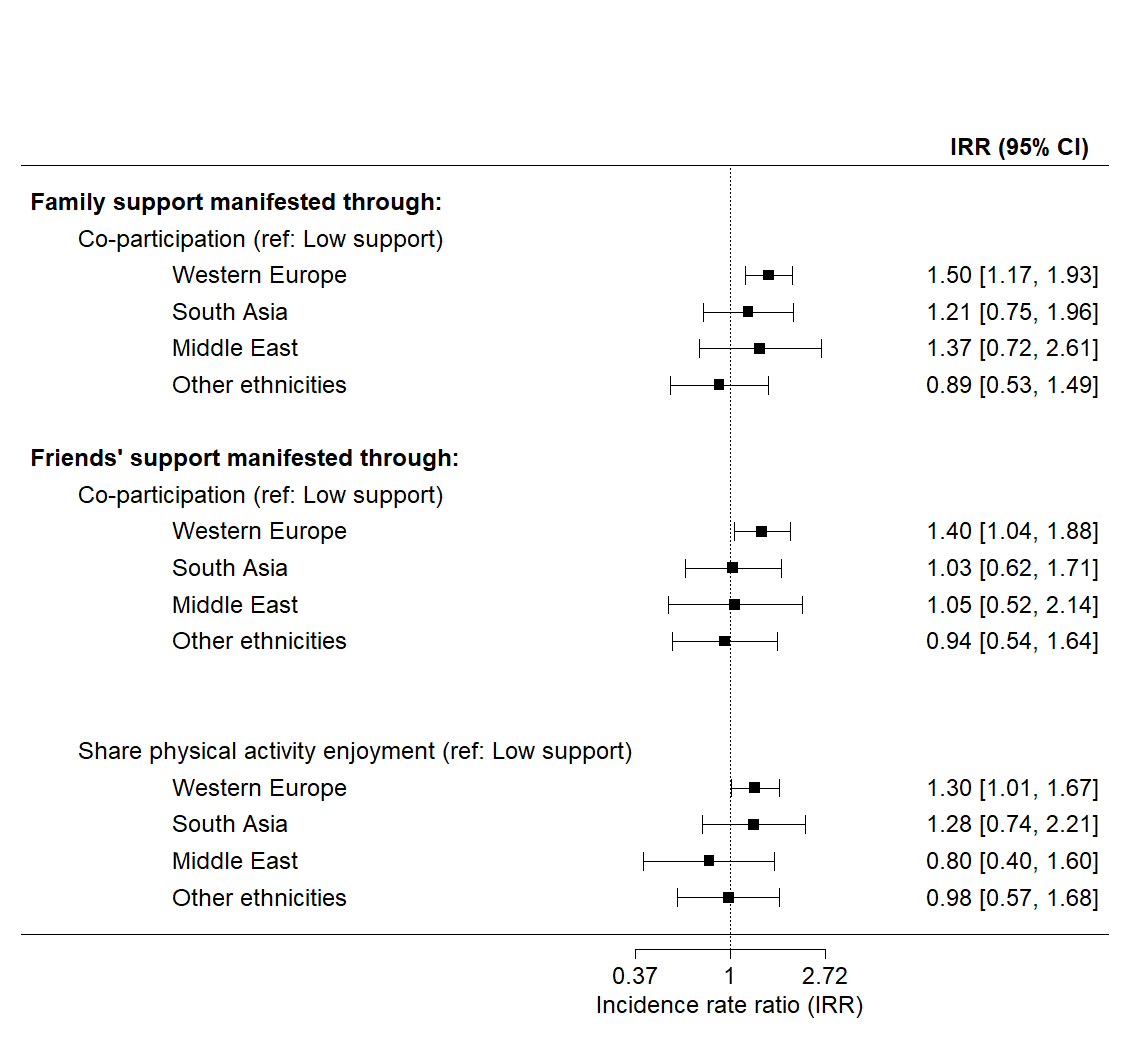

Supplement: Supplementary file 11 — Supplementary Material 11 [file 12889_2023_15507_MOESM11_ESM.tiff]
