# Supplementary material for: Associations between social support and physical activity in postpartum: a Norwegian multi-ethnic cohort study
Source: BMC Public Health. 2023 Apr 17;23:702. doi: 10.1186/s12889-023-15507-z (PMC10111809; doi:10.1186/s12889-023-15507-z)
Supplement: Supplementary file 2 — Supplementary Material 2 [file 12889_2023_15507_MOESM2_ESM.pdf]

Supplementary material 1: Items measuring the participants’ self-reported control over being physically active

13. Think about how things are for you now. Think about all the types of activity. For each statement, state the degree to which you agree/disagree.

	Totally agree						Totally disagree
Whether I am regularly physically active or not, is entirely up to me	☐ 1	☐ 2	☐ 3	☐ 4	☐ 5	☐ 6	☐ 7
If I want to, I would have no problems being regularly physically active	☐ 1	☐ 2	☐ 3	☐ 4	☐ 5	☐ 6	☐ 7
I would have liked to have been regularly physically active, but I’m not really sure if I can manage	☐ 1	☐ 2	☐ 3	☐ 4	☐ 5	☐ 6	☐ 7
I have full control over being regularly physically active	☐ 1	☐ 2	☐ 3	☐ 4	☐ 5	☐ 6	☐ 7
Being regularly physically active is difficult for me	☐ 1	☐ 2	☐ 3	☐ 4	☐ 5	☐ 6	☐ 7
