# Supplementary material for: Associations between social support and physical activity in postpartum: a Norwegian multi-ethnic cohort study
Source: BMC Public Health. 2023 Apr 17;23:702. doi: 10.1186/s12889-023-15507-z (PMC10111809; doi:10.1186/s12889-023-15507-z)
Supplement: Supplementary file 3 — Supplementary Material 3 [file 12889_2023_15507_MOESM3_ESM.pdf]

Supplementary Table 1a: Distribution of habitual physical activity among postpartum women with and without valid data.

	Valid data[1] N=333	Missing[2] N=303
	n (%)	n (%)
Habitual physical activity		
Jogging		
never	304 (93)	267 (89)
1-2 time/week	14 (4)	24 (8)
3 times/week to daily	9 (3)	9 (3)
Biking		
never	305 (93)	281 (93)
1-2 time/week	16 (5)	15 (15)
3 times/week to daily	7 (2)	6 (2)
Fitness center/ strength training		
never	282 (86)	267 (85)
1-2 time/week	41 (12)	35 (12)
3 times/week to daily	7 (2)	11 (3)
Aerobics		
never	296 (90)	284 (94)
1-2 time/week	27 (8)	13 (4)
3 times/week to daily	5 (2)	5 (2)
Dance (jazz, swing, rock)		
never	311 (95)	278 (94)
1-2 time/week	12 (4)	17 (5)
3 times/week to daily	4 (1)	5 (2)
Ball games		
never	324 (98)	299 (99)
1-2 time/week	5 (1)	1 (1)
3 times/week to daily	1 (1)	0 (0)
Swimming		
never	323 (98)	292 (97)
1-2 time/week	5 (2)	8 (2)
3 times/week to daily	0 (0)	1 (1)
Brisk walk		
never	169 (51)	183 (61)
1-2 time/week	60 (18)	44 (15)
3 times/week to daily	104 (31)	74 (24)
Slow walk		
never	36 (11)	49 (17)
1-2 time/week	62 (19)	54 (18)
3 times/week to daily	232 (70)	192 (65)
Other activities		
never	180 (89)	139 (85)

1-2 time/week	11 (5)	9 (6)
3 times/week to daily	12 (6)	15 (9)

[1] Valid moderate-to-vigorous physical activity (MVPA) defined as ≥2 valid MVPA days, where one valid day consisted of ≥19.2 hours of SenseWear Armband (SWA) wear-time and no missing on other variables.

[2] Missing: women not accepting to wear SWA, having <2 valid days of recorded physical activity or missing on other variables.
