# Supplementary material for: Associations between social support and physical activity in postpartum: a Norwegian multi-ethnic cohort study
Source: BMC Public Health. 2023 Apr 17;23:702. doi: 10.1186/s12889-023-15507-z (PMC10111809; doi:10.1186/s12889-023-15507-z)
Supplement: Supplementary file 4 — Supplementary Material 4 [file 12889_2023_15507_MOESM4_ESM.pdf]

Supplementary Table 2. Model comparison of imputed data based on AIC – family support

**Family support**	Model	[*]Akaike information criterion (AIC) for:			
		NB	Zero-inflated NB	Hurdle Poisson	Hurdle NB
Overall support	1	2943.74	2912.59	10674.07	2912.59
	2	2868.27	2822.01	8482.12	2821.99
Encourage PA	1	2944.56	2913.69	11004.94	2913.68
	2	2871.44	2825.83	8572.46	2825.82
Discuss PA	1	2941.24	2908.90	10827.25	2908.90
	2	2867.09	2820.15	8473.68	2820.15
Co-participation	1	2938.86	2905.82	10813.46	2905.82
	2	2862.65	2814.24	8386.23	2814.18
Take over chores	1	2940.71	2908.62	10833.10	2908.61
	2	2865.76	2818.82	8416.26	2818.77
Health benefits talk	1	2937.41	2904.20	10722.26	2904.18
	2	2870.69	2824.24	8490.86	2824.24
Share PA enjoyment	1	2922.30	2906.51	10907.41	2906.42
	2	2870.92	2820.36	8483.24	2819.90

[*]The AIC is based on averaging the AIC for each imputed dataset. For each family support item, model 2 of the hurdle NB had the smallest AIC estimate and was selected. However, the hurdle NB and ZINB were indistinguishable in some cases.
