# Supplementary material for: Associations between social support and physical activity in postpartum: a Norwegian multi-ethnic cohort study
Source: BMC Public Health. 2023 Apr 17;23:702. doi: 10.1186/s12889-023-15507-z (PMC10111809; doi:10.1186/s12889-023-15507-z)
Supplement: Supplementary file 5 — Supplementary Material 5 [file 12889_2023_15507_MOESM5_ESM.pdf]

Supplementary Table 3. Model comparison of imputed data based on AIC – friends' support

**Friend's support**	Model	Akaike information criterion (AIC) for:			
		NB	Zero-inflated NB	Hurdle Poisson	Hurdle NB
Overall support	1	2944.62	2913.65	10993.68	2912.26
	2	2870.42	2822.74	8543.36	2822.74
Offered to do PA together	1	2942.11	2912.85	10986.34	2905.81
	2	2865.97	2816.49	8491.63	2816.38
Encourage PA	1	2943.93	2912.78	10963.98	2912.71
	2	2871.85	2826.04	8573.63	2826.01
Helpful reminders	1	2944.16	2910.26	10966.99	2910.17
	2	2871.77	2825.71	8570.52	2825.70
Co-participation	1	2943.00	2912.15	10964.08	2911.31
	2	2866.76	2818.24	8424.08	2818.22
Health benefits talk	1	2940.85	2907.69	10822.90	2907.67
	2	2871.34	2825.37	8563.05	2825.37
Share PA enjoyment	1	2944.02	2907.46	10961.74	2907.38
	2	2871.48	2824.35	8458.99	2824.31

[*]The AIC is based on averaging the AIC for each imputed dataset. For each friends' support item, model 2 of the hurdle NB model has the smallest AIC estimate and was selected. However, the hurdle NB and ZINB were indistinguishable in some cases.
