# Supplementary material for: Associations between social support and physical activity in postpartum: a Norwegian multi-ethnic cohort study
Source: BMC Public Health. 2023 Apr 17;23:702. doi: 10.1186/s12889-023-15507-z (PMC10111809; doi:10.1186/s12889-023-15507-z)
Supplement: Supplementary file 6 — Supplementary Material 6 [file 12889_2023_15507_MOESM6_ESM.pdf]

Supplementary Table 1b: Distribution of social support from family and friend among women with and without valid data.

	Postpartum		
	Valid data[1]		Missing[2]
	N=333		N=303
Overall family support (mean/SD)	2.9 (1.0)		2.8 (1.0)
Overall family support, n (%)			
low	175 (52.5)		172 (56.8)
high	158 (47.5)		131 (43.2)
Encourage PA, n (%)			
low	89 (26.7)		88 (29.0)
high	244 (73.3)		215 (71.0)
Discuss PA, n (%)			
low	91 (27.3)		89 (29.4)
high	242 (72.7)		214 (70.6)
Co-participate, n (%)			
low	188 (56.5)		184 (60.7)
high	145 (43.5)		119 (39.3)
Take over chores, n (%)			
low	169 (50.8)		162 (53.5)
high	164 (49.2)		141 (46.5)
Health benefits talk, n (%)			
low	100 (30.0)		87 (28.7)
high	233 (70.0)		216 (71.3)
Share PA enjoyment, n (%)			
low	93 (27.9)		99 (32.7)
high	240 (72.1)		201 (67.3)
Overall friends' support (mean/SD)	2.5 (0.9)		2.4 (0.9)
low, n (%)	250 (75.1)		234 (77.2)
high, n (%)	83 (24.9)		69 (22.8)
Offered to do PA together, n (%)			
low	137 (41.1)		150 (49.5)
high	196 (58.9)		153 (50.5)
Encourage PA, n (%)			
low	146 (43.8)		136 (44.9)
high	187 (56.2)		167 (55.1)
Helpful reminders, n (%)			
low	196 (58.9)		176 (58.1)
high	137 (41.1)		127 (41.9)
Co-participation, n (%)			
low	232 (69.7)		224 (73.9)
high	101 (30.3)		79 (26.1)
Health benefits talk, n (%)			

low	150 (45.0)		127 (41.9)
high	183 (55.0)		176 (58.1)
Share PA enjoyment, n (%)			
low	115 (34.5)		134 (44.2)
high	218 (65.5)		169 (55.8)

[1] Valid MVPA data defined as ≥2 valid MVPA days, where one valid day consisted of ≥19.2 hours of SWA wear-time and no missing on other variables.

[2] Missing: women not accepting to wear SWA, having <2 valid days of recoded PA or missing on other variables in the analytic model.

PA: physical activity
