# Supplementary material for: Associations between social support and physical activity in postpartum: a Norwegian multi-ethnic cohort study
Source: BMC Public Health. 2023 Apr 17;23:702. doi: 10.1186/s12889-023-15507-z (PMC10111809; doi:10.1186/s12889-023-15507-z)
Supplement: Supplementary file 7 — Supplementary Material 7 [file 12889_2023_15507_MOESM7_ESM.pdf]

Supplementary Table 1c: Characteristics of women with and without valid data.

	Postpartum		
	Valid data[1]		Missing[2]
	N=333		N=303
Age, years (mean/SD)	30.5 (4.6)		29.3 (4.8)
Body mass index, kg/m² (mean/SD)[d]	25.8 (4.6)		26.4 (5.3)
Time of residence, years (mean/SD)[d]	9.5 (8.1)		9.3 (7.6)
Marital status, n (%)			
Single	10 (3.0)		12 (4.0)
Partner	323 (97.0)		290 (96.0)
Ethnicity, n (%)			
Western Europe	170 (51.1)		102 (33.6)
South Asia	67 (20.1)		89 (29.4)
Middle East	41 (12.3)		53 (17.5)
Other ethnicities*	55 (16.5)		59 (19.5)
Education, n (%)			
Primary school or less	45 (13.5)		49 (16.3)
High school / secondary	110 (33.0)		136 (45.3)
College / University	178 (53.5)		115 (38.4)
Occupation, n (%)[d]			
Elementary occupations and homemakers	86 (25.8)		102 (34.1)
Clerical service and assembly occupation	107 (32.1)		105 (35.1)
Managers and degree occupations	140 (42.1)		92 (30.8)
Parity, n (%)			
Nulliparous	148 (44.4)		146 (48.2)
Parous	185 (55.6)		157 (51.8)

[1] Valid data defined as ≥2 valid MVPA days, where one valid day consisted of ≥19.2 hours of SWA wear-time and no missing on other variables.

[2] Missing: women not accepting to wear SWA, having <2 valid days of recorded physical activity or missing on other variables.

* Other ethnicities: Eastern Europe, East Asia and Africa South of Sahara
