# Supplementary material for: Associations between social support and physical activity in postpartum: a Norwegian multi-ethnic cohort study
Source: BMC Public Health. 2023 Apr 17;23:702. doi: 10.1186/s12889-023-15507-z (PMC10111809; doi:10.1186/s12889-023-15507-z)
Supplement: Supplementary file 8 — Supplementary Material 8 [file 12889_2023_15507_MOESM8_ESM.pdf]

Supplementary Table 4. Associations between family support and MVPA min/day based on complete cases

**Family support**		NB	Zero-inflated NB (ZINB)	Hurdle Poisson	Hurdle NB
**Count part**	Model	IRR (95% CI)	IRR (95% CI)	IRR (95% CI)	IRR (95% CI)
Overall support	1	1.08 (0.93, 1.25)	1.08 (0.95, 1.21)	1.07 (1.05,1.09) **	1.08 (0.95, 1.22)
	2	1.14 (1.00, 1.31) *	1.12 (1.00, 1.25) *	1.11 (1.08,1.13) ***	1.12 (1.01, 1.24) *
Encourage PA	1	1.05 (0.76, 1.45)	1.07 (0.82, 1.39)	1.06 (1.02, 1.11) **	1.07 (0.82,1.39)
	2	1.11 (0.82, 1.51)	1.12 (0.87, 1.43)	1.04 (1.00, 1.08)	1.12 (0.88, 1.43)
Discuss PA	1	1.37 (0.99, 1.89)	1.37 (1.05, 1.79) *	1.37 (1.31, 1.43) ***	1.37 (1.05, 1.79) *
	2	1.42 (1.05, 1.92) *	1.38 (1.09, 1.76) *	1.28 (1.22, 1.34) ***	1.38 (1.09, 1.77) **
Co-participation	1	1.42 (1.07, 1.89) *	1.33 (1.05, 1.68) *	1.31 (1.26, 1.36) ***	1.33 (1.05, 1.68) *
	2	1.51 (1.16, 1.97) **	1.40 (1.14, 1.73) **	1.32 (1.26, 1.37) ***	1.40 (1.14, 1.73) **
Take over chores	1	1.34 (1.00, 1.78) *	1.31 (1.04, 1.65) *	1.30 (1.25, 1.35) ***	1.31 (1.04, 1.65) *
	2	1.39 (1.07, 1.80) *	1.34 (1.09, 1.64) **	1.29 (1.24, 1.34) ***	1.34 (1.09, 1.65) **
Health benefits talk	1	0.66 (0.48, 0.90) **	0.70 (0.54, 0.89) **	0.71 (0.68, 0.74) ***	0.70 (0.55, 0.89) **
	2	0.85 (0.64, 1.14)	0.84 (0.66, 1.05)	0.83 (0.80, 0.86) ***	0.84 (0.67, 1.05)
Share PA enjoyment	1	1.10 (0.80, 1.52)	1.24 (0.96, 1.60)	1.24 (1.19, 1.30) ***	1.24 (0.96, 1.60)
	2	1.17 (0.86, 1.58)	1.26 (1.00, 1.60)	1.24 (1.18, 1.29) ***	1.27 (1.00, 1.60)
**Binary part**				OR (95% CI)	OR (95% CI)
Overall support	1			1.00 (0.74, 1.37)	1.00 (0.74, 1.37)
	2			0.87 (0.63, 1.22)	0.87 (0.63, 1.22)
Encourage PA	1			1.12 (0.56, 2.22)	1.12 (0.56, 2.22)
	2			1.08 (0.52, 2.27)	1.08 (0.52, 2.27)
Discuss PA	1			0.93 (0.48, 1.79)	0.93 (0.48, 1.79)
	2			0.90 (0.44, 1.83)	0.90 (0.44, 1.83)
Co-participation	1			0.61 (0.32, 1.14)	0.61 (0.32, 1.14)
	2			0.58 (0.29, 1.14)	0.58 (0.29, 1.14)
Take over chores	1			0.84 (0.46, 1.53)	0.84 (0.46, 1.53)
	2			0.82 (0.43, 1.56)	0.82 (0.43, 1.56)
Health benefits talk	1			1.52 (0.76, 3.06)	1.52 (0.76, 3.06)

	2			1.03 (0.48, 2.18)	1.03 (0.48, 2.18)
Share PA enjoyment	1			2.33 (1.05, 5.17)	2.33 (1.05, 5.17) *
	2			2.06 (0.88, 4.82)	2.06 (0.88, 4.82)
