# Supplementary material for: Associations between social support and physical activity in postpartum: a Norwegian multi-ethnic cohort study
Source: BMC Public Health. 2023 Apr 17;23:702. doi: 10.1186/s12889-023-15507-z (PMC10111809; doi:10.1186/s12889-023-15507-z)
Supplement: Supplementary file 9 — Supplementary Material 9 [file 12889_2023_15507_MOESM9_ESM.pdf]

Supplementary Table 5. Associations between friends' support and MVPA min/day based on complete cases

**Friends' support**		NB	Zero-inflated NB (ZINB)	Hurdle Poisson	Hurdle NB
**Count part**	Model	IRR (95% CI)	IRR (95% CI)	IRR (95% CI)	IRR (95% CI)
Overall support	1	0.99 (0.84, 1.16)	0.96 (0.84, 1.10)	0.96 (0.94, 0.98) ***	0.96 (0.83, 1.09)
	2	1.09 (0.95, 1.27)	1.04 (0.93, 1.18)	1.06 (1.04, 1.09) ***	1.05 (0.93, 1.18)
Offered to do PA together	1	1.27 (0.95, 1.69)	1.14 (0.89, 1.45)	1.09 (1.05, 1.13) ***	1.11 (0.87, 1.41)
	2	1.39 (1.07, 1.81) *	1.22 (0.99, 1.51)	1.20 (1.15, 1.25) ***	1.22 (0.99, 1.51)
Encourage PA	1	0.88 (0.66, 1.18)	0.87 (0.69, 1.11)	0.87 (0.84, 0.91) ***	0.87 (0.69, 1.11)
	2	1.03 (0.79, 1.34)	0.99 (0.80, 1.23)	0.98 (0.94, 1.02)	0.99 (0.80, 1.23)
Helpful reminders	1	0.90 (0.67, 1.20)	0.87 (0.69, 1.11)	0.88 (0.84, 0.91) ***	0.87 (0.68, 1.10)
	2	0.95 (0.73, 1.24)	0.92 (0.75, 1.14)	0.96 (0.92, 0.99) **	0.92 (0.75, 1.14)
Co-participation	1	1.22 (0.90, 1.66)	1.17 (0.91, 1.50)	1.14 (1.11, 1.20) ***	1.16 (0.90, 1.49)
	2	1.38 (1.04, 1.83) *	1.28 (1.03, 1.60) *	1.29 (1.24, 1.34) ***	1.29 (1.03, 1.61) *
Health benefits talk	1	0.75 (0.57, 1.00)	0.77 (0.61, 0.97) **	0.77 (0.74, 0.80) ***	0.77 (0.61, 0.97) *
	2	0.90 (0.68, 1.19)	0.89 (0.72, 1.11)	0.93 (0.89, 0.97) ***	0.89 (0.72, 1.11)
Share PA enjoyment	1	1.13 (0.84, 1.53)	1.17 (0.92, 1.50)	1.16 (1.11, 1.20) ***	1.18 (0.92, 1.51)
	2	1.10 (0.83, 1.45)	1.16 (0.93, 1.45)	1.26 (1.20, 1.31) ***	1.16 (0.93, 1.45)
**Binary part**				OR (95% CI)	OR (95% CI)
Overall support	1			0.84 (0.60, 1.18)	0.84 (0.60, 1.18)
	2			0.72 (0.51, 1.03)	0.72 (0.51, 1.03)
Offered to do PA together	1			0.43 (0.23, 0.79) **	0.43 (0.23, 0.79) **
	2			0.42 (0.22, 0.80) **	0.42 (0.22, 0.80) **
Encourage PA	1			0.96 (0.53, 1.75)	0.96 (0.53, 1.75)
	2			0.77 (0.40, 1.47)	0.77 (0.40, 1.47)
Helpful reminders	1			0.84 (0.46, 1.56)	0.84 (0.46, 1.56)
	2			0.81 (0.42, 1.58)	0.81 (0.42, 1.58)
Co-participation	1			0.67 (0.33, 1.34)	0.67 (0.33, 1.34)
	2			0.50 (0.24, 1.07)	0.50 (0.24, 1.07)
Health benefits talk	1			1.23 (0.67, 2.26)	1.23 (0.67, 2.26)
	2			0.84 (0.43, 1.66)	0.84 (0.43, 1.66)
Share PA enjoyment	1			1.32 (0.69, 2.54)	1.32 (0.69, 2.54)

	2			1.31 (0.65, 2.65)	1.31 (0.65, 2.65)
