# Supplementary material for: Associations between social support and physical activity in postpartum: a Norwegian multi-ethnic cohort study
Source: BMC Public Health. 2023 Apr 17;23:702. doi: 10.1186/s12889-023-15507-z (PMC10111809; doi:10.1186/s12889-023-15507-z)
Supplement: Supplementary file 12 — Supplementary Material 12 [file 12889_2023_15507_MOESM12_ESM.pdf]

Figure legends

- **Supplementary Figure 1. Directed Acyclic Graph (DAG):** Visualization of the causal assumptions concerning the relationship between social support and moderate-to-vigorous physical activity in postpartum. Unmeasured = Unmeasured confounders. The impact of social support on MVPA in postpartum was found by adjusting for age, ethnicity, parity and education at visit 1 (gestational week 15), and body mass index measured at visit 3 (postpartum). Additionally, we included SenseWear Armband week in postpartum to improve precision in estimates.

- **Supplementary Figure 2. Flowchart:** Flowchart of study participation in the Stork-Groruddalen Study and participants with valid physical activity data recorded by SenseWearTM Armband in postpartum

- **Supplementary Figure 3. Forest plot:** Forest plot shows the impact of support from family or friends on the incidence rate ratio (IRR) of moderate-to-vigorous physical activity with 95% confidence interval by ethnic groups
